# Supplementary material for: A Critical View of the Application of the APEX Software (Aqueous Photochemistry of Environmentally-Occurring Xenobiotics) to Predict Photoreaction Kinetics in Surface Freshwaters
Source: Molecules. 2019 Dec 18;25(1):9. doi: 10.3390/molecules25010009 (PMC7017383; doi:10.3390/molecules25010009)
Supplement: Supplementary file 1 [file molecules-25-00009-s001.zip › 3.2.4_gcc-4.4.0.zip!/3.2.4_gcc-4.4.0/3.2.4_gcc-4.4.0/doc/octave/html/interpreter/Trigonometry.html]

Trigonometry - Untitled


Next: Sums and Products,
Previous: Complex Arithmetic,
Up: Arithmetic


---

### 17.3 Trigonometry

Octave provides the following trigonometric functions where angles are
specified in radians. To convert from degrees to radians multiply by
`pi/180`
(e.g., `sin (30 * pi/180)` returns the sine of 30 degrees). As
an alternative, Octave provides a number of trigonometric functions
which work directly on an argument specified in degrees. These functions
are named after the base trigonometric function with a d suffix. For
example, `sin` expects an angle in radians while `sind` expects an
angle in degrees.

— Mapping Function: **sin** (x)  
> Compute the sine for each element of x in radians.
>
>
>
> **See also:** asin, sind, sinh.

— Mapping Function: **cos** (x)  
> Compute the cosine for each element of x in radians.
>
>
>
> **See also:** acos, cosd, cosh.

— Mapping Function: **tan** (z)  
> Compute the tangent for each element of x in radians.
>
>
>
> **See also:** atan, tand, tanh.

— Mapping Function: **sec** (x)  
> Compute the secant for each element of x in radians.
>
>
>
> **See also:** asec, secd, sech.

— Mapping Function: **csc** (x)  
> Compute the cosecant for each element of x in radians.
>
>
>
> **See also:** acsc, cscd, csch.

— Mapping Function: **cot** (x)  
> Compute the cotangent for each element of x in radians.
>
>
>
> **See also:** acot, cotd, coth.

— Mapping Function: **asin** (x)  
> Compute the inverse sine in radians for each element of x.
>
>
>
> **See also:** sin, asind.

— Mapping Function: **acos** (x)  
> Compute the inverse cosine in radians for each element of x.
>
>
>
> **See also:** cos, acosd.

— Mapping Function: **atan** (x)  
> Compute the inverse tangent in radians for each element of x.
>
>
>
> **See also:** tan, atand.

— Mapping Function: **asec** (x)  
> Compute the inverse secant in radians for each element of x.
>
>
>
> **See also:** sec, asecd.

— Mapping Function: **acsc** (x)  
> Compute the inverse cosecant in radians for each element of x.
>
>
>
> **See also:** csc, acscd.

— Mapping Function: **acot** (x)  
> Compute the inverse cotangent in radians for each element of x.
>
>
>
> **See also:** cot, acotd.

— Mapping Function: **sinh** (x)  
> Compute the hyperbolic sine for each element of x.
>
>
>
> **See also:** asinh, cosh, tanh.

— Mapping Function: **cosh** (x)  
> Compute the hyperbolic cosine for each element of x.
>
>
>
> **See also:** acosh, sinh, tanh.

— Mapping Function: **tanh** (x)  
> Compute hyperbolic tangent for each element of x.
>
>
>
> **See also:** atanh, sinh, cosh.

— Mapping Function: **sech** (x)  
> Compute the hyperbolic secant of each element of x.
>
>
>
> **See also:** asech.

— Mapping Function: **csch** (x)  
> Compute the hyperbolic cosecant of each element of x.
>
>
>
> **See also:** acsch.

— Mapping Function: **coth** (x)  
> Compute the hyperbolic cotangent of each element of x.
>
>
>
> **See also:** acoth.

— Mapping Function: **asinh** (x)  
> Compute the inverse hyperbolic sine for each element of x.
>
>
>
> **See also:** sinh.

— Mapping Function: **acosh** (x)  
> Compute the inverse hyperbolic cosine for each element of x.
>
>
>
> **See also:** cosh.

— Mapping Function: **atanh** (x)  
> Compute the inverse hyperbolic tangent for each element of x.
>
>
>
> **See also:** tanh.

— Mapping Function: **asech** (x)  
> Compute the inverse hyperbolic secant of each element of x.
>
>
>
> **See also:** sech.

— Mapping Function: **acsch** (x)  
> Compute the inverse hyperbolic cosecant of each element of x.
>
>
>
> **See also:** csch.

— Mapping Function: **acoth** (x)  
> Compute the inverse hyperbolic cotangent of each element of x.
>
>
>
> **See also:** coth.

— Mapping Function: **atan2** (y, x)  
> Compute atan (y / x) for corresponding elements of y
> and x. Signal an error if y and x do not match in size
> and orientation.

Octave provides the following trigonometric functions where angles are
specified in degrees. These functions produce true zeros at the appropriate
intervals rather than the small roundoff error that occurs when using
radians. For example:

```
     cosd (90)
          => 0
     cos (pi/2)
          => 6.1230e-17
```

— Function File: **sind** (x)  
> Compute the sine for each element of x in degrees. Returns zero
> for elements where x`/180` is an integer.
>
>
>
> **See also:** asind, sin.

— Function File: **cosd** (x)  
> Compute the cosine for each element of x in degrees. Returns zero
> for elements where `(`x`-90)/180` is an integer.
>
>
>
> **See also:** acosd, cos.

— Function File: **tand** (x)  
> Compute the tangent for each element of x in degrees. Returns zero
> for elements where x`/180` is an integer and `Inf` for
> elements where `(`x`-90)/180` is an integer.
>
>
>
> **See also:** atand, tan.

— Function File: **secd** (x)  
> Compute the secant for each element of x in degrees.
>
>
>
> **See also:** asecd, sec.

— Function File: **cscd** (x)  
> Compute the cosecant for each element of x in degrees.
>
>
>
> **See also:** acscd, csc.

— Function File: **cotd** (x)  
> Compute the cotangent for each element of x in degrees.
>
>
>
> **See also:** acotd, cot.

— Function File: **asind** (x)  
> Compute the inverse sine in degrees for each element of x.
>
>
>
> **See also:** sind, asin.

— Function File: **acosd** (x)  
> Compute the inverse cosine in degrees for each element of x.
>
>
>
> **See also:** cosd, acos.

— Function File: **atand** (x)  
> Compute the inverse tangent in degrees for each element of x.
>
>
>
> **See also:** tand, atan.

— Function File: **asecd** (x)  
> Compute the inverse secant in degrees for each element of x.
>
>
>
> **See also:** secd, asec.

— Function File: **acscd** (x)  
> Compute the inverse cosecant in degrees for each element of x.
>
>
>
> **See also:** cscd, acsc.

— Function File: **acotd** (x)  
> Compute the inverse cotangent in degrees for each element of x.
>
>
>
> **See also:** cotd, acot.
